# Singlet‐Contrast Magnetic Resonance Imaging: Unlocking Hyperpolarization with Metabolism**

**DOI:** 10.1002/anie.202014933

**Published:** 2021-02-11

**Authors:** J. Eills, E. Cavallari, R. Kircher, G. Di Matteo, C. Carrera, L. Dagys, M. H. Levitt, K. L. Ivanov, S. Aime, F. Reineri, K. Münnemann, D. Budker, G. Buntkowsky, S. Knecht

**Affiliations:** ^1^ Helmholtz Institute Mainz GSI Helmholtzzentrum für Schwerionenforschung 64291 Darmstadt Germany; ^2^ Johannes Gutenberg University 55090 Mainz Germany; ^3^ Dept. of Molecular Biotechnology and Health Sciences University of Torino Torino 10126 Italy; ^4^ Technical University of Kaiserslautern 67663 Kaiserslautern Germany; ^5^ Institute of Biostructures and Bioimaging National Research Council of Italy Torino 10126 Italy; ^6^ School of Chemistry University of Southampton Southampton SO17 1BJ Vereinigtes Königreich; ^7^ International Tomography Center Siberian Branch of the Russian Academy of Science Novosibirsk 630090 Russia; ^8^ Novosibirsk State University Novosibirsk 630090 Russia; ^9^ Eduard-Zintl-Institute for Inorganic Chemistry and Physical, Chemistry Technical University Darmstadt 64287 Darmstadt Germany

**Keywords:** hyperpolarization, NMR, parahydrogen, MRI, singlet order

## Abstract

Hyperpolarization‐enhanced magnetic resonance imaging can be used to study biomolecular processes in the body, but typically requires nuclei such as ^13^C, ^15^N, or ^129^Xe due to their long spin‐polarization lifetimes and the absence of a proton‐background signal from water and fat in the images. Here we present a novel type of ^1^H imaging, in which hyperpolarized spin order is locked in a nonmagnetic long‐lived correlated (singlet) state, and is only liberated for imaging by a specific biochemical reaction. In this work we produce hyperpolarized fumarate via chemical reaction of a precursor molecule with *para*‐enriched hydrogen gas, and the proton singlet order in fumarate is released as antiphase NMR signals by enzymatic conversion to malate in D_2_O. Using this model system we show two pulse sequences to rephase the NMR signals for imaging and suppress the background signals from water. The hyperpolarization‐enhanced ^1^H‐imaging modality presented here can allow for hyperpolarized imaging without the need for low‐abundance, low‐sensitivity heteronuclei.

## Graphical abstract

A proton singlet state on fumarate derived from parahydrogen can be broken by enzymatic conversion to malate, which releases hyperpolarized NMR signals. We demonstrate two “out‐of‐phase echo” pulse sequences to convert the *I*_1z_ *I*_2z_ state into net proton magnetization, and show how this can be used for hyperpolarized ^1^H imaging.

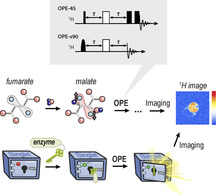

## Introduction

Magnetic resonance imaging (MRI) is a powerful clinical technique most commonly used to produce structural images of the human body from observation of water and fat molecules, which are easily detectable because of their relatively high concentration. Unfortunately, many interesting biomolecular processes occurring in the body involve metabolites at lower concentrations, which precludes MRI‐observation of these biochemical reactions, owing to the inherently low sensitivity of MRI. Recent advances in the field of hyperpolarization‐enhanced nuclear magnetic resonance (NMR) have made it possible to produce metabolites with NMR signal enhancements of 10^4^–10^5^.[^1^, ^2^, ^3^, ^4^, ^5^, ^6^, ^7^, ^8^, ^9^, ^10^, ^11^, ^12^, ^13^, ^14^, ^15^, ^16^] One example of such a hyperpolarization method is parahydrogen induced polarization (PHIP) in which hydrogen gas enriched in the *para* spin isomer is chemically reacted with an unsaturated molecule to generate a product with hyperpolarized ^1^H nuclear spins.[^17^, ^18^, ^19^, ^20^, ^21^, ^22^, ^23^, ^24^, ^25^, ^26^] In a second step, the hyperpolarization can be transferred to other nuclei, for example, ^13^C or ^15^N.[^26^, ^27^, ^28^, ^29^, ^30^, ^31^, ^32^, ^33^] PHIP is renowned for being inexpensive, simple to use, and allows for the production of hyperpolarized substrates with a high repetition cycle.

For a number of reasons, ^1^H would be the ideal nucleus for hyperpolarization‐enhanced MRI:[^34^, ^35^, ^36^]

1. *Sensitivity*—^1^H‐detection is more sensitive than, for example, ^13^C by a factor of ≈16, and ^15^N by a factor of ≈100, if they are polarized to the same degree and are at the same abundance;
2. *Equipment availability*—^1^H probes are readily available in commercial MRI scanners;
3. *Isotopic abundance*—Unlike ^13^C or ^15^N MRI, there is no need for expensive isotopic enrichment of the samples;
4. *Spatial resolution*—Greater response to magnetic field gradients means higher spatial resolution can be obtained than for lower‐γ nuclei.

However, there are some notable drawbacks to hyperpolarized ^1^H MRI:

1. *Rapid relaxation*—Relaxation of hyperpolarized proton signals typically occurs with a characteristic temporal scale on the order of seconds, which is not enough time to perform the complete imaging experiment from sample hyperpolarization to metabolism and detection;
2. *Proton background*—There is a large proton background signal in the body because the natural abundance is ≈100 %, and water and fat molecules are prevalent in the body;
3. *Chemical shift dispersion*—The relatively small chemical shift dispersion can make distinguishing different chemical species a challenge, especially in vivo where broad NMR lines are common.

Owing to these challenges, ^13^C is currently the preferred nucleus for hyperpolarization‐enhanced imaging. The relaxation times are on the order of tens of seconds;^[1]^ the low (1.1 %) natural abundance means there are no significant background signals, and the chemical shift range is an order of magnitude higher than that for protons. One option that has been demonstrated is to store the hyperpolarized magnetization on a heteronucleus, and then transfer it onto a nearby ^1^H spin for signal read‐out.[^37^, ^38^] However, this method still requires isotopic enrichment of the samples and rf probes with a heteronuclear channel.

Singlet‐state NMR (see ref. [39, 40, 41, 42, 43, 44, 45, 46, 47]) offers an alternative possibility to overcome the drawbacks associated with hyperpolarized ^1^H imaging.[^48^, ^49^] When parahydrogen is added to an unsaturated precursor molecule, the protons remain in a nonmagnetic singlet state, as long as they remain chemically and magnetically equivalent. This state is neither directly observable in MRI, nor can it be manipulated by radiofrequency (rf) pulses. Additionally, the proton singlet state is immune to certain relaxation mechanisms, and can have a much longer lifetime compared to proton magnetization.[^39^, ^40^] Thus, the hyperpolarization can be stored in the singlet state until the molecule undergoes a chemical reaction that renders the protons chemically or magnetically inequivalent. This breaks the proton singlet state, and observable hyperpolarized NMR signals are released.

These favourable properties of the singlet state open up new possibilities to perform hyperpolarized ^1^H MRI by injecting a biomolecule supporting a singlet state with a long nuclear spin lifetime, which is converted in vivo to an NMR‐visible substrate. Such an experiment would have the following advantages:

1. Until metabolism of the molecule, the protons relax relatively slowly;
2. The background ^1^H NMR signals of water and fat in the body can be suppressed with rf pulse techniques, while the nonmagnetic singlet state remains unaffected;[^50^, ^51^, ^52^, ^53^]
3. This experiment relies on the appearance of an NMR signal rather than a peak shift, and so is insensitive to the limited chemical shift dispersion.

**Figure 1:**
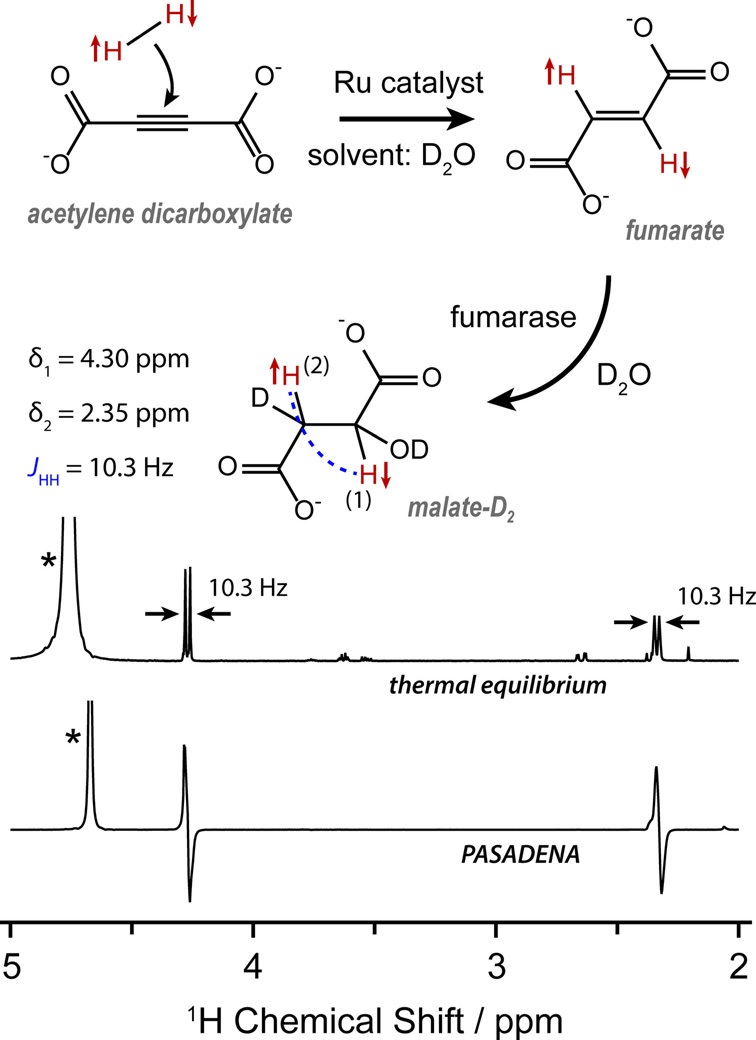
Top: the hydrogenation reaction used to produce fumarate[^15^, ^32^] and the subsequent enzymatic conversion into [3,O‐D_2_]malate (since D_2_O is the solvent). Bottom: ^1^H NMR spectra acquired at a field strength of 11.7 T, showing the difference between a PASADENA spectrum and a thermal equilibrium spectrum of [3,O‐D_2_]malate. The PASADENA spectrum has been vertically scaled down for a clearer comparison. The water peaks (marked by asterisks) are from the residual protons in the solvent, and the shift of water between spectra is a temperature effect.

In this work we demonstrate “singlet‐contrast magnetic resonance imaging” using fumarate, a representative biomolecule. Unlike previous work on this chemical system which used dissolution dynamic nuclear polarization (D‐DNP) to generate singlet order,^[4]^ we produce fumarate by chemically reacting *para*‐enriched hydrogen gas with an acetylene precursor in D_2_O, using a ruthenium *trans*‐hydrogenation catalyst.[^15^, ^32^] Fumarate is a metabolite in the citric acid (Krebs) cycle, and is converted into malate by addition of a water molecule; a reaction catalysed by the enzyme fumarase, and of great importance for hyperpolarization‐enhanced MRI.[^9^, ^10^, ^11^, ^54^, ^55^, ^56^, ^57^] The enzymatic conversion to malate renders the fumarate protons chemically inequivalent. Since the fumarate protons originate from parahydrogen, the malate becomes hyperpolarized, and the resulting enhanced NMR signals are antiphase. These PASADENA (Parahydrogen And Synthesis Allow Dramatically Enhanced Nuclear Alignment) signals can be observed by applying a 45° rf pulse.^[17]^ The formation and metabolism of fumarate are shown in Figure 1, alongside a comparison between PASADENA and thermal equilibrium ^1^H NMR spectra of malate.

Here we perform the enzymatic reaction in D_2_O to produce [3,O‐D_2_]malate (see Figure 1), which will henceforth be referred to as malate‐D_2_ for simplicity. The deuterons are weakly coupled to the protons and are unaffected by the proton rf pulses, so they can be ignored when we consider the spin dynamics. This means we can treat the two protons originating from parahydrogen as an isolated two‐spin system, which is convenient for this proof‐of‐principle demonstration. Using D_2_O as the solvent has the additional and important benefit of extending the fumarate proton *T*_s_, which was measured to be 8 s in H_2_O (see Supporting Information), but is over 40 s in D_2_O.^[32]^

**Figure 2:**
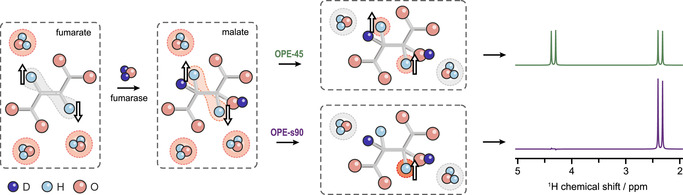
A graphical depiction of hyperpolarized fumarate being converted into malate‐D_2_, followed by the application of OPE‐45 or OPE‐s90 to convert the antiphase proton spin order into in‐phase magnetization, and suppress background signals from water. The white arrows represent different types of spin order, with up‐down arrows in the first two frames indicating the absence of net magnetization on the proton spins. Red and grey shading is used to represent observable and unobservable spin groups, respectively, from different species throughout the experiment. Simulations of the resulting hyperpolarized spectra are shown on the right, using the NMR parameters given in Figure 1. Note that in principle OPE‐45 and OPE‐s90 produce the same integral of the proton signal.

The PASADENA signals from malate‐D_2_ (see Figure 1) are antiphase which precludes most ^1^H imaging techniques, because the application of imaging gradients to spatially encode the magnetization would cause signal cancellation. A pulse sequence is required to convert the two‐spin order into observable, in‐phase magnetization, and additionally suppress signals from background magnetization arising, for instance, from water or fat molecules present in significantly higher concentration. For this purpose, we utilize two versions of the out‐of‐phase echo (OPE) pulse sequence.[^58^, ^59^] In one version, OPE‐45, a hard 45° pulse nonselectively excites the signals, and background magnetization is removed at the end with pulsed field gradients. In the other version, OPE‐s90, a *selective* 90° pulse is applied to the malate‐D_2_ proton resonance at 2.5 ppm, which does not excite background magnetization. The theory of how these two pulse sequences work is given in the Supporting Information. Figure 2 illustrates the core principle of the experiment and how the pulse sequences operate.

## Results and Discussion

### Pulse Sequence Optimization

**Figure 3:**
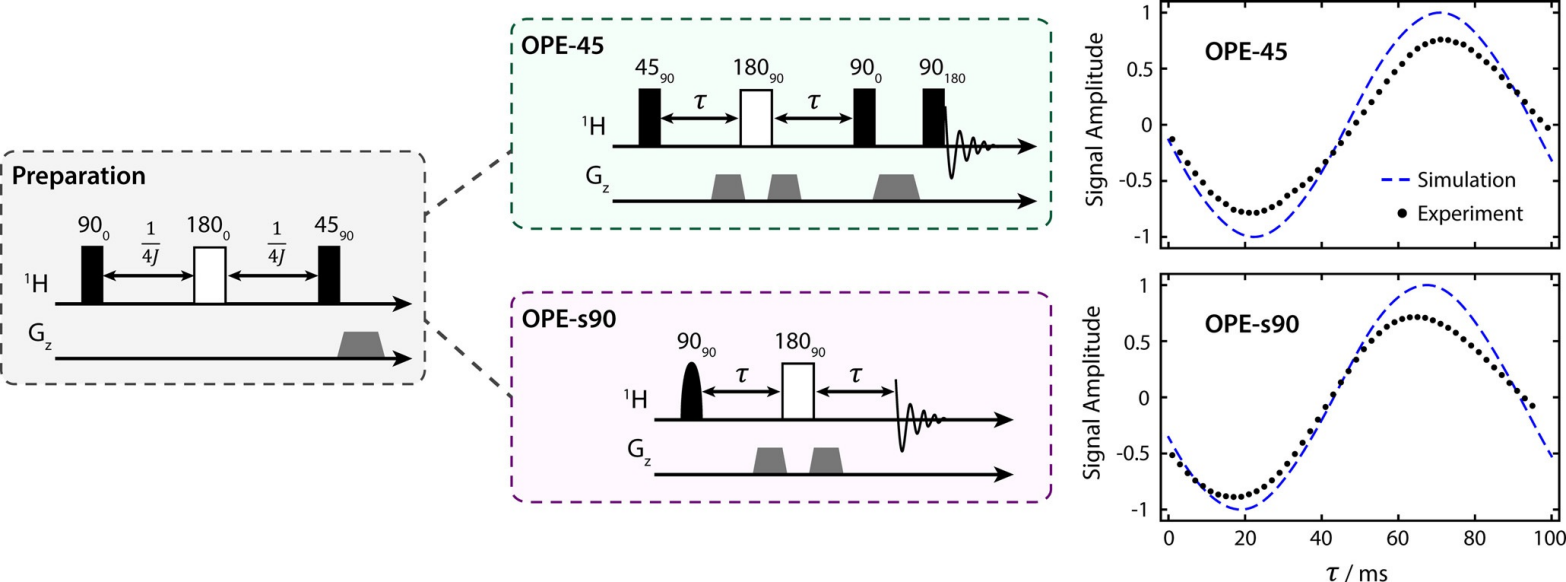
The rf pulse sequences used in this work. Rectangular boxes represent “hard” (non‐selective) pulses, rounded boxes represent “soft” (selective) pulses, and grey trapezoids represent pulsed field gradients. The malate‐D_2_ signal integrals are plotted on the right as a function of the *τ* delay. A simulation of each experiment is shown by the dashed blue line. The simulation and data are normalized to 1, which corresponds to the absolute observable signal if a 45° rf pulse were applied after the preparation sequence. The maximum amplitude of the data is less than 1 because of losses due to relaxation. Error bars have been omitted as they are contained within the plot markers.

To optimise the parameters of the two OPE sequences, experiments were performed on a sample of malate‐D_2_ in D_2_O at thermal equilibrium (i.e. not generated from parahydrogen). A preparation rf pulse sequence known as the Sarkar sequence^[60]^ was applied to convert the thermal equilibrium *I*_1z_+*I*_2z_ spin order into *I*_1z_ *I*_2z_ spin order between the protons, to mimic the initial density operator in an experiment using parahydrogen. Immediately after, an OPE sequence was applied. This experiment was repeated many times, with the delay in the OPE varied to find the optimum value. The pulse sequences and results for both the OPE‐45 and OPE‐s90 are shown in Figure 3.

### PHIP Shuttling Experiments

**Figure 4:**
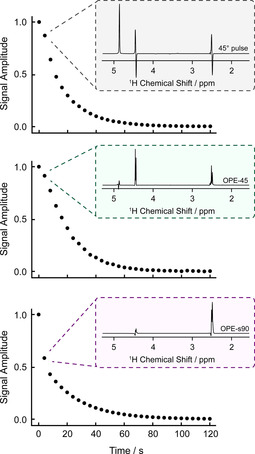
Integral plots showing decay of the hyperpolarized malate‐D_2_ signals after shuttling of hyperpolarized fumarate into a solution of fumarase enzyme in D_2_O. The three experiments each used a different rf pulse method to reveal the malate‐D_2_ signal after the fumarate landed in the enzyme solution: a 45° pulse, OPE‐45, or OPE‐s90 (as drawn in Figure 3). The rf pulse sequences were applied every 4 s to reveal the malate‐D_2_ molecules that formed during that time. The spectrum of the second data point is shown for each of the sequences. For the 45° pulse experiment data, the left‐half of the 4.3 ppm peak was integrated.

To demonstrate the pulse sequences in hyperpolarized NMR experiments we used the following procedure: (1) bubble *para*‐enriched hydrogen gas into the precursor solution to produce hyperpolarized fumarate; (2) pneumatically shuttle the sample into an NMR tube containing fumarase in D_2_O held in an 11.7 T magnet; (3) apply either OPE‐45, OPE‐s90, or a 45° pulse every 4 s and detect the resulting NMR signal. The results are shown in Figure 4. Each pulse sequence destroys most or all of the hyperpolarized spin order in malate‐D_2_ with each application, but the singlet order for fumarate molecules is unaffected. We observe persistent NMR signals for approximately 1 minute, which is possible because new molecules of malate‐D_2_ form between the application of each pulse sequence. The water signal is significantly attenuated in the OPE‐45 spectra, and virtually absent from the OPE‐s90 spectra.

The malate‐D_2_^1^H polarization level was estimated to be 20 % in the OPE‐s90 experiment. This was determined by summing the integrals of the 2.35 ppm peak during the hyperpolarized ^1^H signal decay and comparison with the thermal equilibrium signal integral after the hyperpolarization had fully decayed. We were unable to determine the ^1^H polarization level in the OPE‐45 experiment because the proton signal was not visible in the thermal equilibrium spectrum, but we were able to set a lower bound of 10 % assuming a signal‐to‐noise ratio (SNR) of 1 in the thermal equilibrium spectrum.

### Imaging Results

**Figure 5:**
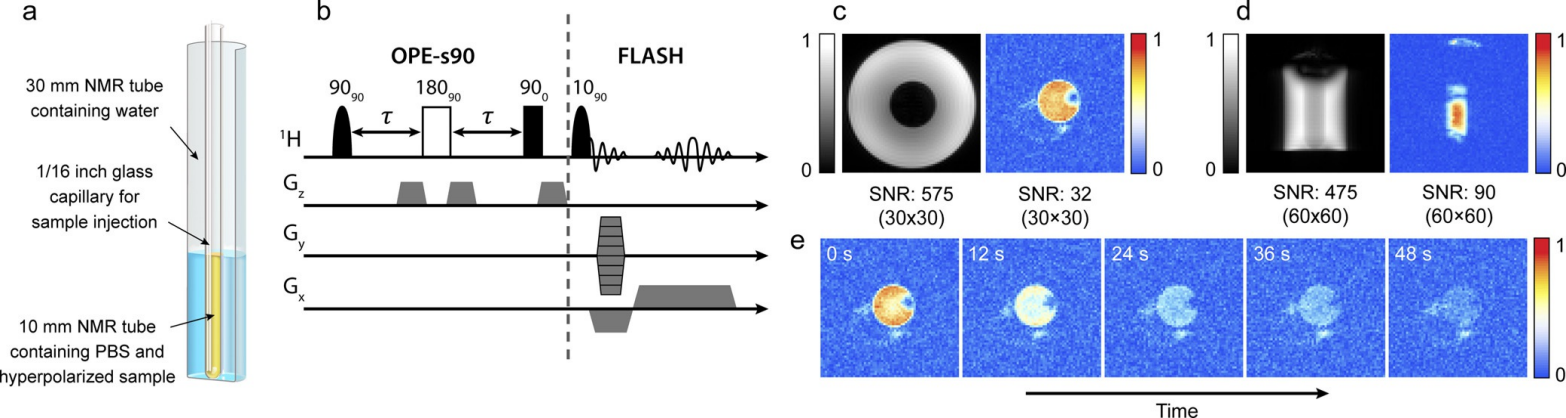
(a) A schematic of the imaging phantom. (b) The pulse sequence used to acquire hyperpolarized ^1^H images. (c,d) A comparison between the hyperpolarized and thermal equilibrium ^1^H images, with the hyperpolarized images acquired following the procedure described in the text. (e) A time series of hyperpolarized ^1^H images. The receiver gain was set to 101 for hyperpolarized image acquisition and 1 for thermal equilibrium image acquisition, which gives a factor of 2 difference in signal‐to‐noise ratio (as discussed in the Supporting Information).

To demonstrate singlet‐contrast imaging we acquired images of a hyperpolarized reaction mixture in a 10 mm NMR tube surrounded by H_2_O. This is shown in Figure 5 a. The imaging was performed in a 7 T magnetic field. The 10 mm NMR tube initially contained fumarase in a deuterated phosphate buffer solution (PBS) at pH 7 (optimal for this reaction^[61]^). The precursor solution was hydrogenated with parahydrogen and then injected into the 10 mm NMR tube for imaging. Further experimental details are given in the Materials and Methods section (see Supporting Information).

To image the hyperpolarized malate that formed from the metabolism, we applied OPE‐s90 to selectively excite the malate protons, immediately followed by a 90° rf pulse to return the magnetization back to the *z*‐axis, and then applied a fast low‐angle shot (FLASH) sequence^[62]^ with centric reordering to acquire a 64×64 pixel image. The complete imaging sequence is shown in Figure 5 b. The sequence was repeated every 12 s to acquire a train of images as the metabolism progressed. For comparison, we also show nonselective ^1^H images acquired using the same FLASH sequence after the hyperpolarized signals had fully relaxed. The metabolites in the 10 mm tube are at such a low concentration that they are not visible in the thermal equilibrium images, but a large water background signal is present. The PHIP experiment was performed twice to acquire axial and sagittal images, and the first image from each acquisition train is shown in Figure 5 c,d alongside the nonselective thermal equilibrium images. The train of images acquired in the axial‐orientation experiment are shown in Figure 5 e. The receiver gain was set to 101 for hyperpolarized experiments, and 1 for thermal equilibrium experiments, and this translates to a factor of 2 difference in SNR.

The singlet‐contrast MRI experiments shown in Figure 5 demonstrate that despite the low concentration of the metabolites present (approximately 10^3^ times lower than the concentration of H_2_O molecules), this technique can be used to suppress the water background signal and image metabolic flux. A FLASH sequence with centric reordering was used here as a convenient way to utilize the hyperpolarized magnetization generated by the OPE sequences, as only a small fraction of magnetization is used for the acquisition of each line in k‐space. However, other imaging techniques^[63]^ such as rapid acquisition with relaxation enhancement (RARE)^[64]^ or echo planar imaging (EPI)^[65]^ could be utilized for this experiment. In the current work, no slice selection was applied. This can be readily achieved in the case of OPE‐45 where slice selection can be done in a standard way in the imaging part of the sequence, but is more complex for the case of OPE‐s90 which uses frequency‐selective excitation pulses, as the slice‐selection gradient necessarily produces a large frequency distribution, making frequency selection challenging. It would however be possible to use a slice selective 180 refocusing pulse in the OPE sequence.

In our experiments, OPE‐45 and OPE‐s90 show similar transfer efficiency of *I*_1z_ *I*_2z_ spin order into magnetization, and both sequences have the same theoretical efficiency. OPE‐45 relies on pulsed field gradients and rf pulse phase selection to suppress background signals, whereas OPE‐s90 additionally suppresses background signals due to the frequency selectivity of its excitation pulses. On the other hand, the implementation of the frequency‐selective pulse introduces some additional experimental challenges (e.g. requiring frequency drift of less than tens of hertz between experiments). It should be noted that alternative pulse sequences such as those in the Only ParaHydrogen Spectroscopy (OPSY) family[^50^, ^51^, ^52^, ^53^] can be utilized to generate in‐phase magnetization from antiphase spin order, but these methods are not explored in this work.

A previous hyperpolarized ^1^H imaging experiment^[49]^ utilized in‐phase magnetization generated from the PASADENA signal under *J*‐coupling evolution during the echo time of the imaging scheme. In Singlet‐Contrast MRI, the hyperpolarization is stored in a long‐lived singlet state before imaging, and the method additionally overcomes some of the limitations of that approach; it is more general, as it can be combined with any imaging scheme without synchronizing the delays to the couplings of the hyperpolarized molecule. This allows one to easily acquire images of the hyperpolarized signal at different points in time, which makes real‐time tracing of metabolic processes possible. Additionally, it does not require that the background and hyperpolarized ^1^H signals have different relaxation properties for background suppression.^[34]^

Experiments in this work were performed in D_2_O to extend the lifetime of hyperpolarized spin order, and to produce a spin system with just two proton nuclei in the product molecule ([3,O‐D_2_]malate). Application in biological systems means working in the presence of H_2_O, which reduces the fumarate singlet‐state lifetime. The proton *T*_s_ was measured in a protonated phosphate buffer solution to be 8 s (see Supporting Information), which presents a challenge for applications of this particular molecular system; the hyperpolarized fumarate should be prepared in D_2_O, and only mixed with H_2_O at the point of delivery to minimize signal losses. As an alternative, it has been shown that other molecules can support proton singlet states that are relatively long‐lived in protonated solvents.^[66]^ We note that the 8 s *T*_s_ was measured on [1‐^13^C]fumarate, and fluctuating dipolar coupling to the nearby ^13^C spin is an additional source of relaxation that will not be present in the unlabelled molecules used for singlet‐contrast imaging. The conversion to malate occurring in a protonated solvent also leads to the formation of fully protonated malate, a three‐spin system. In order to convert the initial parahydrogen‐derived three‐spin order into observable in‐phase magnetization, the pulse sequence evolution (*τ*) delays need to be modified, and an overall lower transfer efficiency can be expected. We discuss the three‐spin case further in the Supporting Information and provide the optimal theoretical *τ* delays.

A similar experiment to what has been shown here was demonstrated with DNP‐polarized fumarate, to show that long‐lived spin states can be populated via D‐DNP.^[4]^ In this work we have used PHIP to hyperpolarize the proton singlet state, and show that this type of experiment can be used for imaging. A comparison of PHIP with D‐DNP as polarization sources is relatively straightforward for experiments in which the ^13^C spins in fumarate are hyperpolarized; the polarization levels can be compared by measuring the ^13^C magnetization at the point of delivery. A similar comparison is more subtle for this proton‐enhanced experiment. The fumarate protons support four states: a singlet state and three triplet states. The distribution of population amongst these four states determines the maximum malate signal intensity at the point of detection. In the PHIP experiment the aim is to fully populate the singlet state by starting from *para*‐enriched hydrogen, which would lead to a relative malate signal intensity of 1. In the D‐DNP experiment the polarization process depletes the singlet state, which would lead to a relative malate signal intensity of −1/3, as discussed in the Supporting Information, as well as in ref. [4]. Beyond the highest achievable signal enhancement, PHIP stands out as being significantly less expensive than D‐DNP, and is able to produce boluses of hyperpolarized material at a much higher turnover rate.

## Conclusion

In conclusion, we have demonstrated a novel type of hyperpolarized ^1^H imaging experiment, in which nuclear hyperpolarization is locked in a long‐lived singlet state until liberation by a chemical/biological process. We used *para*‐enriched hydrogen gas to hyperpolarize the proton singlet state in the biomolecule fumarate, and the signals were released by enzymatic conversion to malate. In our experiments, up to 20 % proton spin polarization was observed on malate. The released signals are antiphase when observed directly after applying an rf pulse which complicates imaging, and so we employed two pulse sequences, OPE‐45 and OPE‐s90, for converting the antiphase spin order into in‐phase magnetization. By adding pulsed field gradients for coherence filtering, we show that background signals from the protons in the water solvent can be effectively suppressed. We have demonstrated the method by acquiring images of hyperpolarized fumarate‐to‐malate metabolism over the course of a minute using OPE‐s90, with effective suppression of the water background signals. The relatively short proton singlet lifetime in water (8 s) will likely limit this specific molecular system to studying samples with high metabolic flux. We expect this imaging method to be extended to alternative PHIP systems,^[67]^ or other nuclear spin species, for example, ^15^N or ^13^C singlet pairs,[^68^, ^69^, ^70^, ^71^, ^72^, ^73^] which are known to be long‐lived in aqueous solution.

## Conflict of interest

The authors declare no conflict of interest.

## Supporting information

As a service to our authors and readers, this journal provides supporting information supplied by the authors. Such materials are peer reviewed and may be re‐organized for online delivery, but are not copy‐edited or typeset. Technical support issues arising from supporting information (other than missing files) should be addressed to the authors.

Supplementary
