# Supplementary material for: Singlet‐Contrast Magnetic Resonance Imaging: Unlocking Hyperpolarization with Metabolism
Source: Angew Chem Int Ed Engl. 2021 Feb 11;60(12):6791–8. doi: 10.1002/anie.202014933 (PMC7986935; doi:10.1002/anie.202014933)
Supplement: Supplementary file 1 — Supplementary [file ANIE-60-6791-s001.pdf]

Angewandte Chemie
Eine Zeitschrift der Gesellschaft Deutscher Chemiker

Supporting Information

Singlet-Contrast Magnetic Resonance Imaging: Unlocking Hyperpolarization with Metabolism**

J. Eills, E. Cavallari, R. Kircher, G. Di Matteo, C. Carrera, L. Dagys, M. H. Levitt, K. L. Ivanov, S. Aime, F. Reineri, K. Münnemann, D. Budker, G. Buntkowsky,* and S. Knecht**

anie_202014933_sm_miscellaneous_information.pdf

Contents

1. Materials and methods
2. Pulse sequence theory
3. Simulations of the OPE sequences for a three-spin system
4. Enzyme concentration dependence
5. Receiver gain calibration
6. Dissolution DNP vs PHIP
7. T_S measurement

1. Materials and Methods

All chemicals were purchased from Sigma Aldrich. A solution of 50 mM disodium acetylene dicarboxylate, 100 mM sodium sulphite and 7 mM ruthenium catalyst [RuCp*(CH_3CN)$_3$]PF_6 in D_2O was prepared by dissolving the solids by heating and sonication. The sodium sulphite was added to increase the rate of reaction as discussed in Ref 31 of the main text. The solution was filtered using a polytetrafluoroethylene (PTFE) syringe filter (Whatman UNIFLO) with 0.22 µm diameter pores to remove any residual solid particles. This precursor solution was degassed (i.e. the oxygen was removed) by bubbling helium through for 5 minutes, and this was used for all experiments.

The spectroscopy experiments were performed in an 11.7 T magnet, in a 5 mm ^{1}H-^{13}C dual resonance probe, using an AVANCE III console. The imaging experiments were performed in a 7 T vertical bore magnet, using a quadrature proton imaging probe.

Parahydrogen at >98% enrichment was generated using an Advanced Research Systems parahydrogen generator for all hyperpolarization experiments.

Pulse-sequence optimisation
To generate the [3,O-D_2]malate sample for the pulse sequence optimization experiments, 300 mM disodium fumarate was dissolved in D_2O. Subsequently, 25 µL of fumarase was added to this sample to catalyse the formation of [3,O-D_2]malate. This sample was kept in a 5 mm NMR tube overnight at room temperature for the enzyme to degrade and used without further alteration.

Hyperpolarized ^{1}H spectroscopy experiments
A low-pressure/vacuum 5 mm NMR tube (Wilmad Glass) containing 500 µL of the precursor solution was held in an oil bath maintained at 80°C. *Para*-enriched hydrogen gas was bubbled through the solution at 7 bar for 1 minute. Following this, the solution was pneumatically shuttled through PTFE tubing (1.6 mm O.D., 0.5 mm I.D.) using helium gas at 7 bar for 7 s into a second 5 mm NMR tube. This tube contained a solution of 5 µL fumarase in 145 µL D_2O, and was held in an 11.7 T magnet at 25°C. After shuttling, pulse sequences were applied, and the signal was acquired every 4 s.

Hyperpolarized ^{1}H imaging experiments
For the imaging experiments, a 10 mm NMR tube containing 25 µL fumarase in a 50 mM deuterated phosphate buffer solution at pH 7 with a total volume of 2.25 mL was held in the centre of a 30 mm NMR tube containing deionised water. This phantom setup was held in a 7 T vertical-bore imaging magnet.

A low-pressure/vacuum 5 mm NMR tube (Wilmad Glass) containing 900 µL of the precursor solution was held in an oil bath maintained at 80°C. *Para*-enriched hydrogen gas was bubbled through the solution at 8 bar for 30 seconds. Following this, the pressure was released and the solution was extracted into a syringe for injection into the phosphate buffer solution in the magnet. The solution was injected through a 1/16 inch O.D. PTFE line, terminating in a 1/16 inch glass capillary in the detection region. This was followed by a few seconds of pushing air through the capillary to ensure efficient mixing of the reaction solution with the PBS/enzyme solution. Immediately following this, the imaging sequences were applied, and the signal was acquired every 12 s. The final volume in the 10 mm NMR tube was approximately 3 mL since some sample is lost during the transfer, mostly as droplets in the PTFE line.

2. Pulse sequence theory

The nuclear spin Hamiltonian for the two spin system is

$$H = J_{12} I_{1z} I_{2z} + \omega_1 I_{1z} + \omega_2 I_{2z}$$

where I_{1z} represents the z-angular momentum operator for spin 1, J_{12} is the scalar coupling between spins 1 and 2, and ω_i is the Larmor frequency of spin i. We can neglect *J* couplings to the deuterium nucleus, and additionally neglect the flip-flop components of the proton-proton *J* coupling interaction, which is valid when $|\omega_1 - \omega_2| \gg J_{12}$.

The fumarate protons which originated from parahydrogen are in a singlet state, which can be written as a sum of Cartesian product operators:

$$\rho_{\text{singlet}} = \frac{\mathbb{1}}{4} - \left(I_{1\text{x}}I_{2\text{x}} + I_{1\text{y}}I_{2\text{y}} + I_{1\text{z}}I_{2\text{z}}\right)$$

where $\mathbb{1}$ is the identity operator and will be dropped henceforth. Upon conversion to malate-D_2, the protons become chemically inequivalent and the singlet state is no longer an eigenstate. For an ensemble of malate molecules formed at different time points under the condition $|\omega_1 - \omega_2| > J_{12}$, the initial density operator is, to a good approximation,

$$\rho_0 = I_{1\text{z}}I_{2\text{z}}$$

Applying a 45° rf pulse produces the antiphase spectral pattern shown in Fig. 1 in the main text. We now consider how the OPE (Out-of-Phase Echo) pulse sequences convert this two-spin order to in-phase magnetization and suppress the water background.

1. *OPE-45*

After applying a 45° pulse about the y-axis to both spins, the density operator is:

$$\rho_1^{(45)} = \frac{1}{2}(I_{1\text{z}}I_{2\text{z}} + I_{1\text{z}}I_{2\text{x}} + I_{1\text{x}}I_{2\text{z}} + I_{1\text{x}}I_{2\text{x}})$$

with the superscript denoting the OPE-45 sequence used. Applying a spin echo generates the operator:

$$\rho_2^{(45)}(\tau) = \frac{1}{4}\left(2\cos(2\pi J\,\tau)\,(I_{1\text{z}}I_{2\text{x}} + I_{1\text{x}}I_{2\text{z}}) + 2(I_{1\text{z}}I_{2\text{z}} + I_{1\text{x}}I_{2\text{x}}) + \sin(2\pi J\,\tau)(I_{1\text{y}} + I_{2\text{y}})\right)$$

and hence, by setting $\tau = {}^{1}/_{4J}$:

$$\rho_2^{(45)}\left(\frac{1}{4J}\right) = \frac{1}{2}(I_{1\text{z}}I_{2\text{z}} + I_{1\text{x}}I_{2\text{x}}) + \frac{1}{4}(I_{1\text{y}} + I_{2\text{y}})$$

which corresponds to in-phase magnetization on both spins, and additional unobservable double-quantum terms.

A 90° pulse about the x-axis returns the in-phase magnetization to the z axis, giving:

$$\rho_3^{(45)} = \frac{1}{2}\left(I_{1\text{y}}I_{2\text{y}} + I_{1\text{x}}I_{2\text{x}}\right) + \frac{1}{4}(I_{1\text{z}} + I_{2\text{z}})$$

A pulsed field gradient is applied along z, which serves to dephase both double-quantum terms, as well as signals originating from magnetization, but leaves the $I_{1\text{z}}$ and $I_{2\text{z}}$ operators unaffected.

To understand how signals originating from magnetization are suppressed by this method, we consider the relevant operators. The Hamiltonian and initial density operator are

$$H^{(1)} = \omega_1 I_{1\text{z}}$$

$$\rho_0^{(1)} = I_{1\text{z}}$$

where the superscript denotes single-spin order. The 45° pulse about the y-axis generates

$$\rho_1^{(1)} = \frac{1}{\sqrt{2}}(I_{1\text{z}} + I_{1\text{x}})$$

and the spin echo produces:

$$\rho_2^{(1)} = \frac{1}{\sqrt{2}}(I_{1\text{z}} - I_{1\text{x}})$$

The 90° pulse about the x-axis converts this into:

$$\rho_3^{(1)} = \frac{1}{\sqrt{2}}\left(I_{1\text{y}} - I_{1\text{x}}\right)$$

which is suppressed by the pulsed field gradient.

2. *OPE-s90*

After applying a selective 90° pulse along the y-axis on-resonance with spin 1, the density operator is

$$\rho_1^{(s90)} = I_{1x} I_{2z}$$

Following the application of the spin echo, the density operator is

$$\rho_1^{(s90)}(\tau) = -\cos(2\pi J \tau)\, I_{1x} I_{2z} - \frac{1}{2}\sin(2\pi J \tau)\, I_{1y}$$

and setting $\tau = {}^{1}/_{4J}$ gives

$$\rho_1^{(s90)}\left(\frac{1}{4J}\right) = -\frac{1}{2} I_{1y}$$

which corresponds to in-phase magnetization on spin 1.

If the initial 90° pulse selectively excites spin 1, and we assume a perfect spin echo, the water background signal is not excited.

Note that both OPE-45 and OPE-s90 produce the same observable signal magnitude.

3. Simulations of the OPE sequences for a three-spin system

When considering applying the presented method in a solvent of H_2O rather than D_2O, the malate produced by the fumarase enzyme would contain an additional proton originating from water. This third spin needs to be considered when calculating the optimal timings for the OPE. We show below the predicted polarization for a three system using the chemical shifts and *J*-couplings of the three malate protons determined at a field of 11.7 T in a pH 7 buffer solution. Figure S1 (top) the selective excitation of two malate protons with the chemical shifts $\delta_1 = 2.36$ ppm (which originates from the catalytic reaction with pH_2) and the proton at $\delta_1 = 2.63$ ppm which originates from the enzymatic conversion of fumarate to malate. In this situation only the nucleus originating from parahydrogen acquires net polarization. When using OPE-45, Figure S1 (bottom), both spins originating from parahydrogen ($\delta_1 = 2.36$ and $3 = 4.27$) acquire net magnetization which for some durations of the OPE cancel each other out.

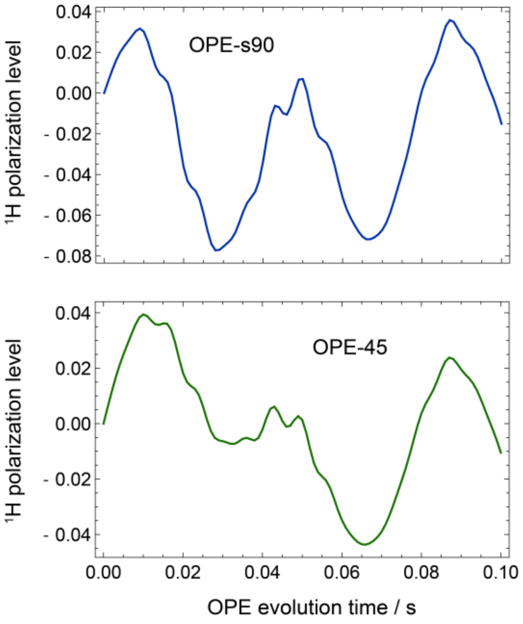

Figure S1: Simulation of the polarization generated by OPE-45 (top) and by OPE-s90 (bottom) for varied τ delay. The simulation intensities are normalized with 1 corresponding to unity polarization on all three proton spins. The *J*-couplings used are $J_{12} = 15.6$, $J_{13} = 10.3$ and $J_{23} = 3.25$, and the field was taken to be 11.7 T.

4. Enzyme concentration dependence

We have studied the rate of fumarate to malate conversion for a given concentration of fumarase enzyme by taking thermal equilibrium ^{1}H NMR spectra during metabolic flux. A reaction to produce a sample of fumarate was carried out in the same

way as described in the Materials and Methods in the main text, using normal hydrogen gas. A 150 µL aliquot of this solution was injected into an NMR tube containing 50 mM phosphate buffer solution at pH 7 in D_2O with fumarase (1.5, 5, 25 µL) to give a total solution volume of 600 µL. The NMR tube was shaken to mix the liquid, and put into an 11.7 T NMR magnet for signal acquisition every 5 s using 10° flip angle pulses. The results for the three experiments are shown in Fig. S2. Note that the 5 µL enzyme experiment is the equivalent of the solution used for the imaging experiments.

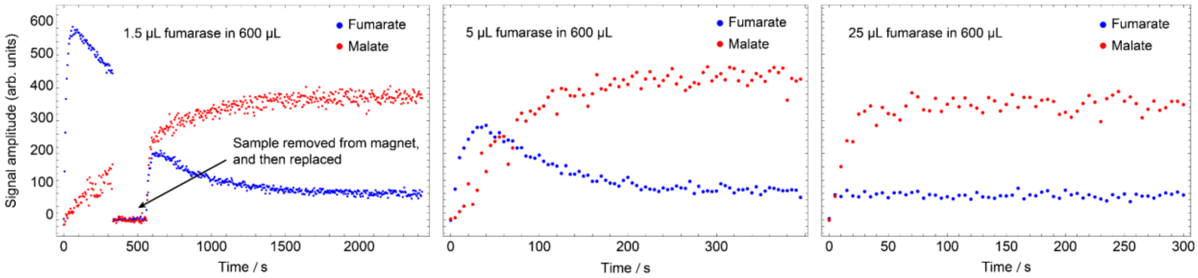

Figure S2: ^{1}H NMR signal intensities of fumarate and malate after adding 150 µL fumarate reaction solution to 450 µL phosphate buffer solution at pH 7 with a varied volume of fumarase.

5. Receiver gain calibration

In order to determine the influence of the receiver gain of the imager on the signal to noise of the obtained images, two FLASH images of a 1 mm slice of water in the 30 mm NMR tube were taken with receiver gain of 1 and 101 (see Fig. S3). The SNR of both images differed by a factor of 1.83. Thus we report a value of 2 as the scaling factor between the hyperpolarized and background MRI images in the main text.

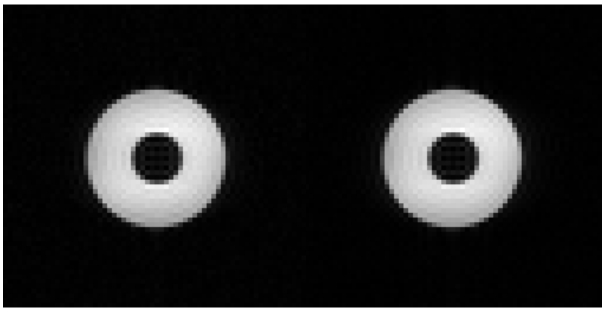

Figure S3: Comparison of two thermal equilibrium MRI images of a 1 mm slice of the phantom used for the experiments in the main text, taken at receiver gain 101 (left) and receiver gain 1 (right).

6. Dissolution DNP vs PHIP

In the main text we make a comparison between the use of PHIP (ParaHydrogen Induced Polarization) and D-DNP (Dissolution Dynamic Nuclear Polarization) as hyperpolarization sources for Singlet-Contrast MRI (Magnetic Resonance Imaging). We discussed that there are four states of the fumarate proton pair: a singlet state and three triplet states. These states are shown in Fig. S4, along with the characteristic relaxation time constants T_1 and T_S. Population imbalances between the singlet and triplet manifolds relax to thermal equilibrium with the time constant T_S, and population imbalances within the triplet manifold relax to thermal equilibrium with the time constant T_1. The following discussion assumes the population of the triplet manifold is spread equally between the three triplet states, which is true to a good approximation after three T_1 periods pass during the experiment; n.b. importantly, this does not necessarily also mix population between the singlet and triplet manifolds, since T_S can be much greater than T_1. The detectable malate signal after enzymatic conversion and application of either OPE-45 or OPE-s90 is proportional to $(4P_S - 1)/3$, where P_S is the population of the fumarate singlet state, which can take a value $0 \leq P_S \leq 1$, and the total population of all four states always sums to 1. In the PHIP experiment we aim to fully populate the singlet state (i.e. produce a sample of fumarate for which $P_S = 1$), which would lead to a relative malate signal of 1. In a D-DNP experiment, the fumarate protons can be fully polarized in the triplet states, i.e. the singlet state is depleted, which corresponds to $P_S = 0$, and leads to a relative malate signal of -1/3.

Polarization via D-DNP polarizes the fumarate protons into either the $|T_{+1}\rangle$ or $|T_{-1}\rangle$ state, and the discussion above assumes that the population redistributes evenly within the three triplet states. This is likely to be a good assumption for most cases, since proton T_1 times are generally on the order of seconds. However, if the enzymatic conversion to malate were to be

performed before any relaxation between the triplet states had occurred, the achievable malate signal intensity would again be 1.

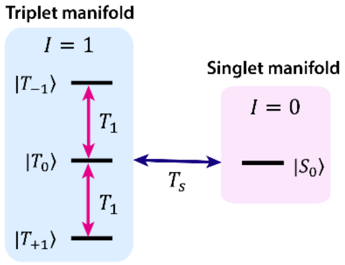

Figure S4: An illustration of the singlet and triplet states of the fumarate proton pair, with the relaxation time constants shown.

7. T_S measurement

The singlet lifetime (T_S) measurement was performed on a 500 µL sample of 50 mM [1-^{13}C]fumarate disodium salt in a phosphate buffer solution at pH 7. The sample was degassed by bubbling nitrogen gas through it for 10 min. The NMR experiments were performed in an 11.7 T magnet at 20°C.

To measure the singlet lifetime, a pulse sequence was used to: (1) convert the thermal equilibrium Zeeman polarization on the protons into singlet order; (2) allow a variable delay for spin relaxation; (3) convert the proton singlet order into ^{13}C magnetization for signal readout. The first component is the magnetisation-to-singlet (M2S) block which is followed by a delay (τ), and the final component is the singlet-to-heteronuclear-magnetisation (S2hM) block. This M2S-τ-S2hM pulse sequence was repeated with a varied τ delay. This method is described in more detail in Ref. [S1], and here we show just the results in Fig. S5. The ^{13}C spectra corresponding to the data points in the figure were collected using 4 transients for each, with repetition delay of 90 s.

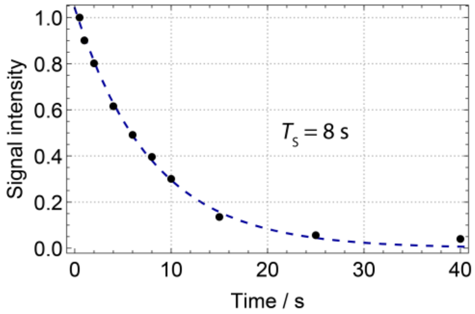

Figure S5: The ^{13}C signal intensity from a series of M2S-τ-S2hM experiments with varied singlet order decay time τ. Data points are shown by black dots, and a monoexponential fit of the form e^{-t/T_S} with T_S set to 8 s is shown by the blue dashed line.

References

(S1) Ripka, B.; Eills, J.; Kouřilová, H.; Leutzsch, M.; Levitt, M. H.; Münnemann, K. Hyperpolarized Fumarate via Parahydrogen. *Chem. Commun.* **2018**, *54* (86), 12246–12249. https://doi.org/10.1039/C8CC06636A.
